# An automated deep learning method and novel cardiac index to detect canine cardiomegaly from simple radiography

**DOI:** 10.1038/s41598-022-18822-4

**Published:** 2022-08-25

**Authors:** Yeojin Jeong, Joohon Sung

**Affiliations:** Genome & Health Data Lab, School of Public Health, Seoul National University, Seoul, Korea

**Keywords:** Diagnosis, Medical imaging, Cardiology, Mathematics and computing

## Abstract

Since most of degenerative canine heart diseases accompany cardiomegaly, early detection of cardiac enlargement is main priority healthcare issue for dogs. In this study, we developed a new deep learning-based radiographic index quantifying canine heart size using retrospective data. The proposed “adjusted heart volume index” (aHVI) was calculated as the total area of the heart multiplied by the heart’s height and divided by the fourth thoracic vertebral body (T4) length from simple lateral X-rays. The algorithms consist of segmentation and measurements. For semantic segmentation, we used 1000 dogs’ radiographic images taken between Jan 2018 and Aug 2020 at Seoul National University Veterinary Medicine Teaching Hospital. The tversky loss functions with multiple hyperparameters were used to capture the size-unbalanced regions of heart and T4. The aHVI outperformed the current clinical standard in predicting cardiac enlargement, a common but often fatal health condition for small old dogs.

## Introduction

The global population of dogs was estimated to be around 900 million in 2013, and 20% of them cohabitate with humans^1^. Cohabitating dogs are often regarded as family members, with growing vigilance on their healthcare. With improved care and increased life span, it is estimated that approximately 10% of dogs represented in primary care veterinary practices have heart disease^2^.

Unlike humans, where coronary heart diseases are predominant, myxomatous mitral valve disease (MMVD) is the most common type of heart disease in dogs, with its prevalence increasing markedly with age. About 75 ~ 80% of small-breed dogs (< 15 kg) over 13 years of age are reported to have degenerative heart diseases^2^. The MMVD is characterized by progressive deformation of the valve structure, resulting in mitral valve regurgitation (MR). The progression of MR results in left heart overload, with signs of left atrial and left ventricular enlargement (LAE and LVE)^3,4^. Since cardiac enlargement ensues pharmacological intervention to prevent fatal heart failure, early detection of cardiac enlargement is a priority healthcare issue for dogs.

The American College of Veterinary Internal Medicine Veterinarian (ACVIM) guideline for staging MMVD^2^ is widely used for diagnosing cardiac enlargement. In the ACVIM consensus guideline, vertebral heart score (VHS)^5^ larger than 11.5 from lateral thoracic radiograph is suggested as radiographic evidence of cardiomegaly alone without echocardiographic measurements. VHS is an index of normalized heart size to body size using mid-thoracic vertebrae for adjustment. For measuring VHS, the longest axis and its perpendicular axis of the cardiac silhouette from a simple canine chest X-ray are summed and then divided by the length of the mid-thoracic vertebral body, starting at the cranial edge of T4. Because of its relative simplicity, the VHS is the most widely used index of cardiac enlargement, at the cost of involving efforts and possible measurement errors.

We propose a new automated cardiac index for dogs to improve the VHS index, adjusted heart volume index (aHVI). The deep learning (DL) algorithms automate the measures and enable two-dimensional measurement of the heart area. The purpose of this study is 1) to develop a method that automatically measures and a new cardiac index from a simple X-ray and 2) to compare the performance of a new method versus the current standard method in predicting clinically proven cardiomegaly.

## Material and methods

### Data sources

The images and clinical information were collected from Seoul National University Veterinary Medicine Teaching Hospital (SNU-VMTH) between January 2018 to August 2020. For all participants, informed consent was obtained by owners. All radiographs were taken with dog in right lateral recumbency and included radiologic reports made by veterinary radiologists at SNU-VMTH.

**Figure 1:**
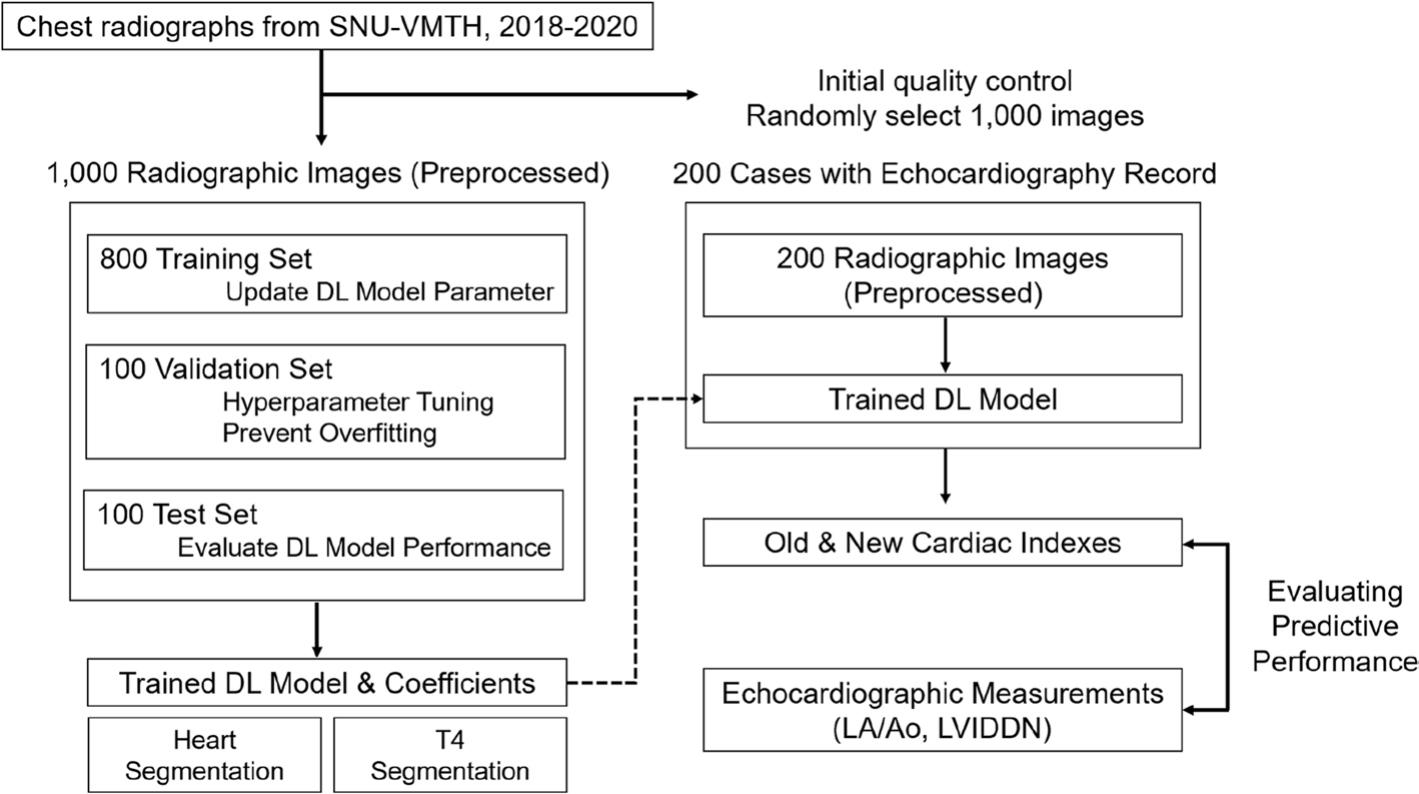
An overall process of developing a new cardiac index of adjusted heart volume index (aHVI). After the quality check, one thousand images were randomly divided into 800 training, 100 validation, and 100 test sets to develop a deep learning model. In the training stage, segmentation, measurements, and optimization were conducted. The performance of aHVI from the trained model was compared with the VHS (vertebral heart score, the current standard, manual measure) against the echocardiographic measurement of cardiomegaly (combined LA/Ao and LVIDDN) using 200 independent data.

A total of 1000 radiographs (1000 dogs) with complete information were randomly selected to develop a radiographic index. Additional 200 images (191 dogs) with concurrent echocardiographs were collected to compare the diagnostic performance of new and conventional indices in detecting echocardiography-confirmed cardiomegaly. The entire workflow is shown in Fig. 1.

After an initial quality check, image data were selected; inadequately positioned or exposed radiographs were excluded by manual inspection of veterinary radiologists. Some disease conditions that interrupt heart margin were excluded, including pleural effusion, overlying mediastinal nodules, and lung mass superimposed over the heart. Clinical information such as sex, age, breed, radiologic report, and manually measured VHS was also collected.

### Image preprocessing and ground truth establishment

All images were center cropped with Pillow library in Python and resized to 256 × 256. Contrast Limit Adaptive Histogram Equalization (CLAHE)^6^ was applied using the OpenCV library^7^ in Python to mitigate variability in exposure level and enhance the contrast of images. Veterinary radiologists in SNU-VMTH manually generated contour labels of heart and T4 vertebrae body with the “labelme opensource tool”^8^. Experts’ labels were used as ground truth to train the semantic segmentation.

### Improved attention U-net

#### Focal Tversky loss

For semantic segmentation, the Dice score coefficient (DSC) is widely used to assess segmentation performance. The 2-class DSC variant for class $c$ is expressed in Eq. (1), where ${g}_{ic}\in \left\{\mathrm{0,1}\right\}$ and ${p}_{ic}\in \left[\mathrm{0,1}\right]$ represent the ground truth label and predicted label, respectively. The total number of pixels in an image is denoted by $N$. The $\epsilon$ provides numerical stability to prevent division by zero. The linear Dice loss (DL) is defined a minimization of the overlap between the prediction and ground truth^9^.

1 $$DSC_{c} = \frac{{\mathop \sum \nolimits_{i = 1}^{N} p_{ic} g_{ic} + \epsilon }}{{\mathop \sum \nolimits_{i = 1}^{N} p_{ic} + g_{ic} + \epsilon }}$$

One of the limitations of the DL is that it equally weights false positive (FP) and false negative (FN) detections. For our model, the segmentation maps for heart required high recall to accurately detect heart contour, while the segmentation maps for T4 needed to penalize false positive detections more strongly to prevent over-estimation of T4 length. The Tversky similarity index is a generalization of the DSC which allows for flexibility in balancing FP and FNs (Eq. (2)):

2 $$TI_{c} = \frac{{\mathop \sum \nolimits_{i = 1}^{N} p_{ic} g_{ic} + \epsilon }}{{\mathop \sum \nolimits_{i = 1}^{N} p_{ic} g_{ic} + \alpha \mathop \sum \nolimits_{i = 1}^{N} p_{{i\overline{ c} }} g_{ic} + \beta \mathop \sum \nolimits_{i = 1}^{N} p_{ic} g_{{i\overline{ c} }} + \epsilon }}$$

where, $p_{ic}$ is the probability that pixel $i$ is of the lesion class $c$ and $p_{{i\overline{ c} }}$ is the probability pixel $i$ is of the non-lesion class $\overline{c}$. The same is true for $g_{ic}$ and $g_{{i\overline{ c} }}$, respectively. Hyperparameters $\alpha$ and $\beta$ can be tuned to shift the emphasis to false positive and false negative detections. The Tversky index is adapted to a loss function (TL) by minimizing $\mathop \sum \limits_{c} 1 - TI_{c}$^10^. In case of $\alpha = \beta = 0.5$, TI simplifies to the DSC. If $\alpha > 0.5,$ TI weights more emphasis on minimizing FN predictions, while setting $\beta$ larger than 0.5 weights more emphasis on minimizing FP predictions.

In practice, DL struggles to segment small region of interest (RoI) as they do not contribute to the loss significantly. To mitigate this problem, the authors of improved attention U-Net paper^11^ proposed the focal Tversky loss function (FTL), which is parameterized by hyperparameter $\gamma$ to control between easy background and hard RoI training examples. The focal parameter exponentiates the cross-entropy loss to focus on hard classes detected with lower probability^12^. FTL is defined as (Eq. (3)):

3 $$FTL_{c} = \mathop \sum \limits_{c} \left( {1 - TI_{c} } \right)^{1/\gamma }$$

When $\gamma >1$, the loss function focuses more on less accurate predictions that have been misclassified. Authors observed the best performance with $\gamma = \frac{4}{3}$. However, they found that using FTL as loss function for all layers over-suppressed FTL when the model is close to converge. So they recommended to train intermediate layers with FTL but supervised the last layer with the Tversky loss to provide a strong signal and mitigate sub-optimal convergence. In this paper, we adapted recommended architecture of improved attention U-Net, but changed values of $\alpha$ and $\beta$ to accurately segment heart and T4 regions.

#### Network architecture

The improved attention U-Net^11^ is based on U-Net^13^, which is composed of a contracting path to extract locality features and an expansive path, to resample the image maps to combine high-resolution local features with low-resolution global features and encourage more semantically meaningful outputs.

However, at the deepest stage of encoding where the network has the richest possible feature representation, spatial details tend to get lost in the high-level output maps. This makes it difficult to reduce false detections for small objects that show large shape variability^14^. So the attention gates (AG) is added to vanilla U-Net architecture to identify relevant spatial information from low-level feature maps and propagate it to the decoding stage. With additive attention gate, input features are scaled with attention coefficients to propagate relevant features to the decoding layer output. The coarser gating signal provides a contextual information while spatial regions from the input features provide locality information. Feature map is then resampled by bilinear interpolation. Details for attention-gated U-Net are explained in^14^.

#### Segmentation model development

**Figure 2:**
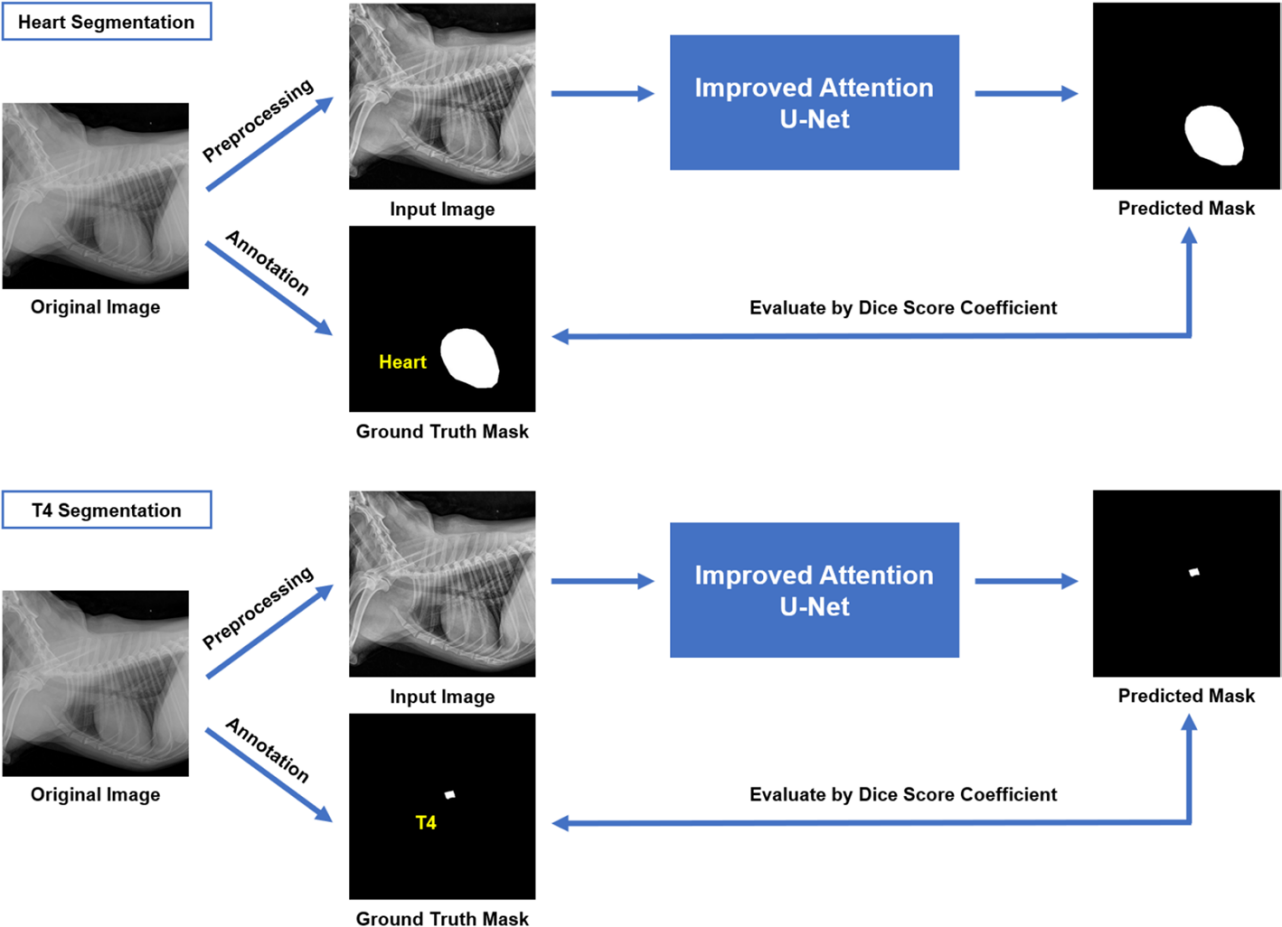
Schematic representation of improved attention U-Net used in semantic segmentation of heart and T4 on canine thoracic radiography. The improved attention U-Net^11^ receives both preprocessed input radiographic images and ground truth mask for the heart (top) and T4 (bottom). Both Networks are trained using Tversky loss. Outputs from the networks are predicted masks of heart and T4. Performance of the network is evaluated by dice score coefficient, recall and, precision between ground truth mask and predicted mask.

The segmentation DL model was developed based on improved attention U-Net which uses focal Tversky loss function^11^. The improved attention U-Net outperformed other segmentation algorithms for multiple imbalanced regions of interest (RoI). Since canine heart (area and diameter) and T4 body (length) were substantially different in size and measurement unit, we trained segmentation models separately with different hyperparameters $\alpha$ for Tversky loss (Fig. 2).

Dataset was randomly split into 800–100-100 train-validation-test sets. The model receives both input image and corresponding ground truth mask contracts the input image by convolution operation to extract locality features and expands the contracted input image to resample the image maps with contextual information. The model uses soft attention gates and injects the contraction path with the multi-scale input image pyramid to reduce measurement errors for DL.

#### Development of novel canine cardiac index, the adjusted heart volume index (aHVI)

**Figure 3:**
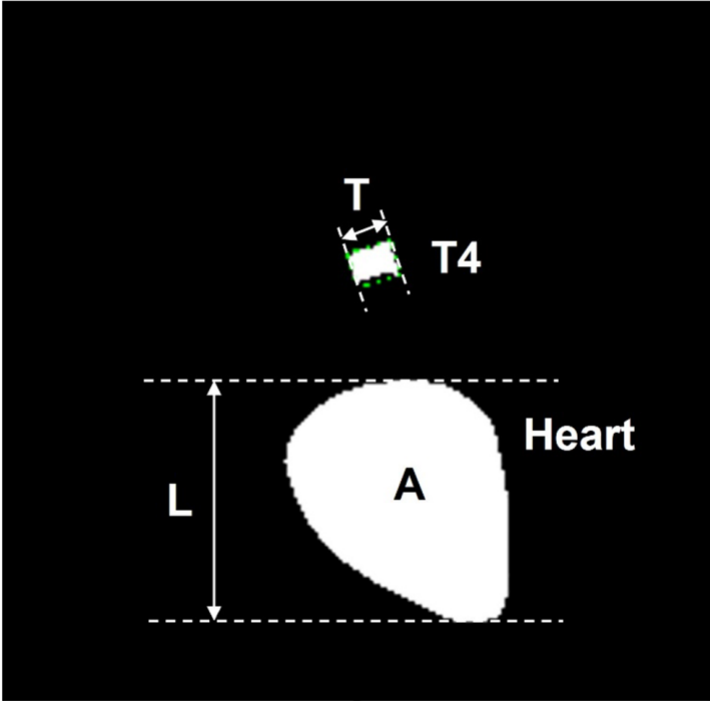
Analysis of binary mask. Area (A) and height (L) of heart and length of T4 (T) are measured. Height of the heart (L) was calculated by the coordinate value of the heart region at its radiographic position. T4 length (T) was measured as width of “minimum area rectangle” of T4 body.

With two binary masks, heart area defined by the experts and one predicted by the DL model, we measured the heart area, heart height, and T4 length using the OpenCV library^15^. The heart's height was measured as the vertical length of the contour from the radiography. The length of the T4 body was measured as the width of the “minimum-area rectangle” surrounding the vertebral body. The description of binary mask analysis is shown in Fig. 3.

We adapted the volumetric quantification methods for echocardiography that multiply the area and diameters^16^. For allometric scaling of heart volume according to the size of dogs, we used T4 length since the earlier study has shown that vertebral body length has an excellent linear correlation with the axial lengths of the heart^5^. The formula for aHVI used in this study is as follows:

$${\text{aHVI}} = ({\text{Area of Heart * Height of Heart) }}/\left( {\text{T4 Length * 10,000}} \right)$$

#### Statistical analysis and diagnostic measures of model development

Dice evaluated segmentation accuracy for heart and T4 score coefficient, precision, and recall between the predicted mask and ground truth mask of 100 test images. Independent 200 data with both X-ray and echocardiography records were used to compare the predictive performance of VHS and aHVI in detecting echocardiography-confirmed cardiomegaly. Echocardiographic LA:Ao ratio in the right-sided short-axis view in early diastole (LA/Ao) over 1.6 was regarded as left atrial enlargement, and left ventricular internal diameter in diastole, normalized for body weight (LVIDDN) over 1.7 was considered as the gold-standard of left ventricular enlargement, according to the ACVIM consensus guideline^2^. Receiver operating characteristics (ROC) analysis was used to compare the diagnostic performance of aHVI and VHS. The aHVI value with the highest difference between true-positive rate and false-positive rate was used as an optimal cutoff value. As recommended by the official guideline, a VHS of 11.5 was used as a cutoff. Each test's sensitivity, specificity, and F1 scores were computed at the cutoff point. We applied standard diagnostic measures of deep learning models including sensitivity, specificity, F1 score, recall and precision. For segmentation models, we measured Pearson’s and Spearman’s correlation coefficients between the ground truth and predicted segments. The beta coefficients of the linear regression model between the ground truth and model prediction were also added as a measure of model performance.

## Results

### Characteristics of participating dogs

A total of 1000 images from 1000 dogs (mean age $\pm$ standard deviation, 9.7 years $\pm$ 4.2; 505 female, 89.2% small breeds) were used for DL model establishment. The model was trained with 800 images and validated and tested with 100 images. Additional 200 data with concurrent simple radiography and echocardiography came from 200 dogs (mean age 10.7 years $\pm$ 3.4; 87 female) (Table 1). Numbers in parentheses are percentages rounded to one decimal place.

**Table 1:** General characteristics of the participating dogs.

Characteristic	DL model development data			Echocardiography validation data (*n* = 200)	Total (*n* = 1200)
	Training set (*n* = 800)	Validation set (*n* = 100)	Test set (*n* = 100)		
**Age (years)**					
Mean	9.7	10.3	8.9	10.7	9.9
Standard Deviation	4.2	4.2	4.5	3.4	4.1
**Sex**					
Male	387 (48.4)	56 (56)	52 (52)	91 (51.1)	586 (49.7)
Female	413 (51.6)	44 (44)	48 (48)	87 (48.9)	592 (50.3)
**VHS**					
Mean	10.2	10.4	10.1	10.6	10.3
Standard Deviation	0.9	1.0	1.0	1.0	0.9
Cardiomegaly suspected (Radiologically)*	375 (46.9)	63 (63)	43 (43)	156 (78)	637 (53.1)
Small Breed Dog	710 (88.8)	91 (91)	91 (91)	170 (96.0)	1062 (90.2)

*Radiographs with report of “possible cardiomegaly” or “rule out mitral valve insufficiency (R/O MVI)”.

### Segmentation model development

The hyperparameter values with the best test performance were chosen for each segmentation DL model. The model with the highest recall was chosen for heart segmentation, and the model with the highest precision was chosen for T4 segmentation. A comparison of tuned hyper-parameters is shown in Table 2.

**Table 2:** Hyperparameters used for segmentation DL models.

Type	Heart segmentation network	T4 body segmentation network
Hyperparameter α for Tversky Loss	0.7	0.2
Hyperparameter γ for Focal Tversky Loss	4/3 (adapted from reference^11^)	
Optimizer	Adam^23^ (Learning rate 0.001, Momentum 0.9)	

Other hyper-parameters setting is listed in Table 3.

**Table 3:** Hyperparameters used for segmentation DL models.

Type	Heart segmentation network	T4 body segmentation network
Network architecture	Improved Attention U-Net^11^	
Data split	Training 800, Validation 100, Test 100	
Input dimension	(512, 512, 1)	
Output dimension	(512, 512, 1)	
Intermediate layer loss function	Focal Tversky Loss	
Final layer loss function	Tversky Loss	
Batch size	16	
Epochs	100	
Callbacks	Reduce Learning Rate on Plateau (factor = 0.1, patience = 15, minimum learning rate = 1e-6, Monitor validation loss) Early Stopping (Stop at epoch with maximum validation DSC, patience = 15)	
Evaluation metrics	Dice score, Recall, Precision	

**Figure 4:**
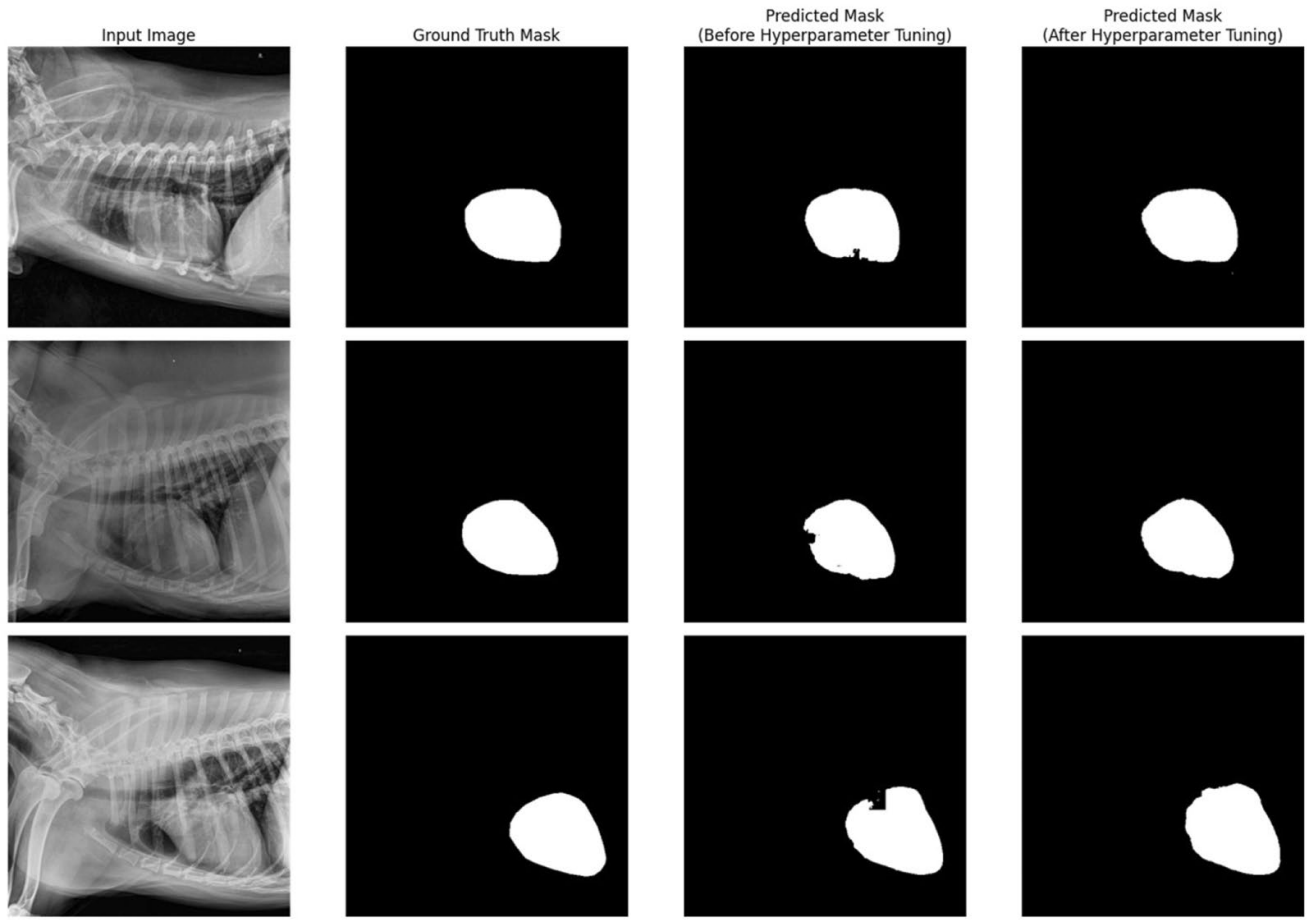
Input images, ground truth masks, and predicted masks for heart segmentation model with default and optimized hyperparameter setting.

**Figure 5:**
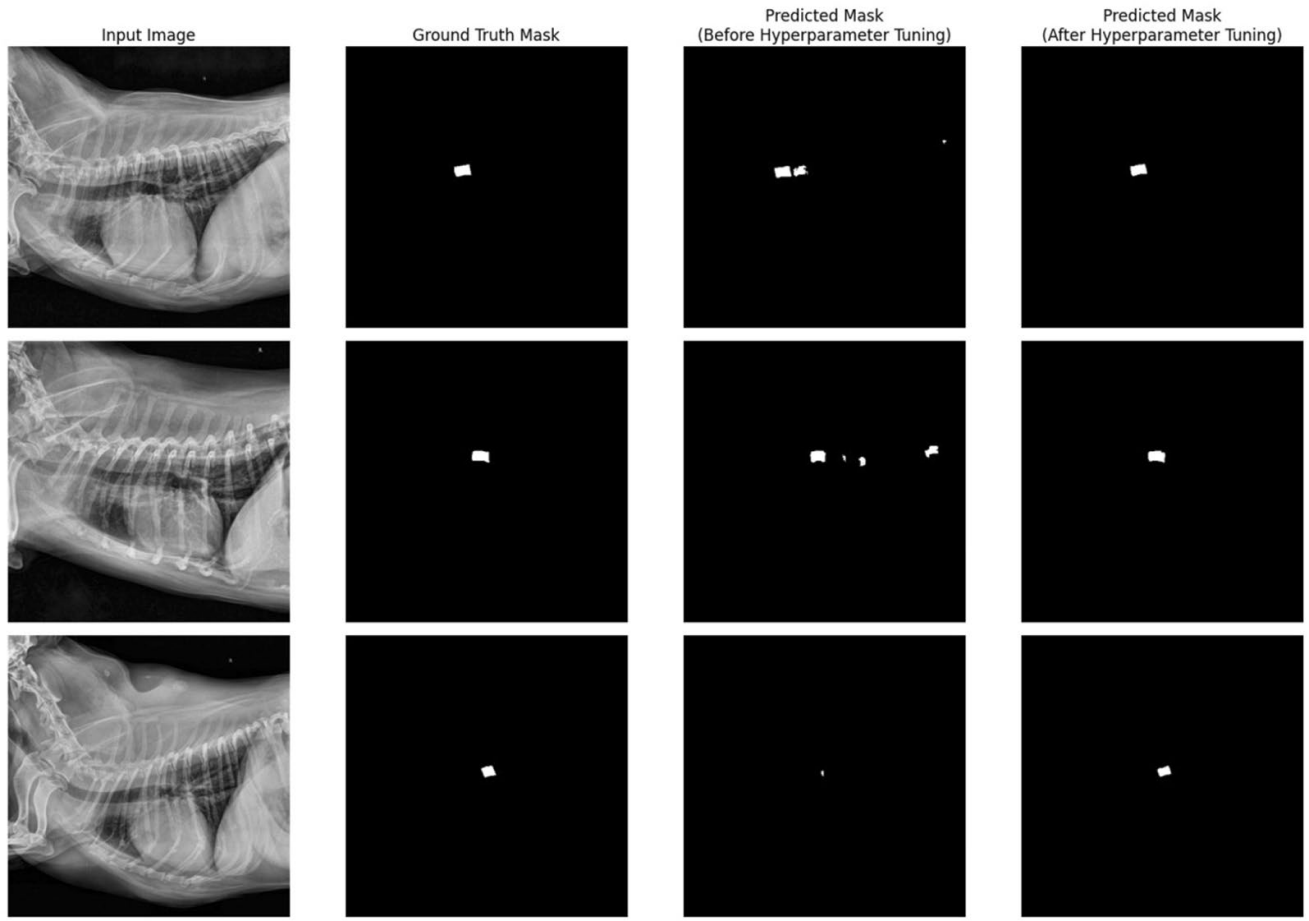
Input images, ground truth masks, and predicted masks for T4 body segmentation model with default and optimized hyperparameter setting.

**Figure 6:**
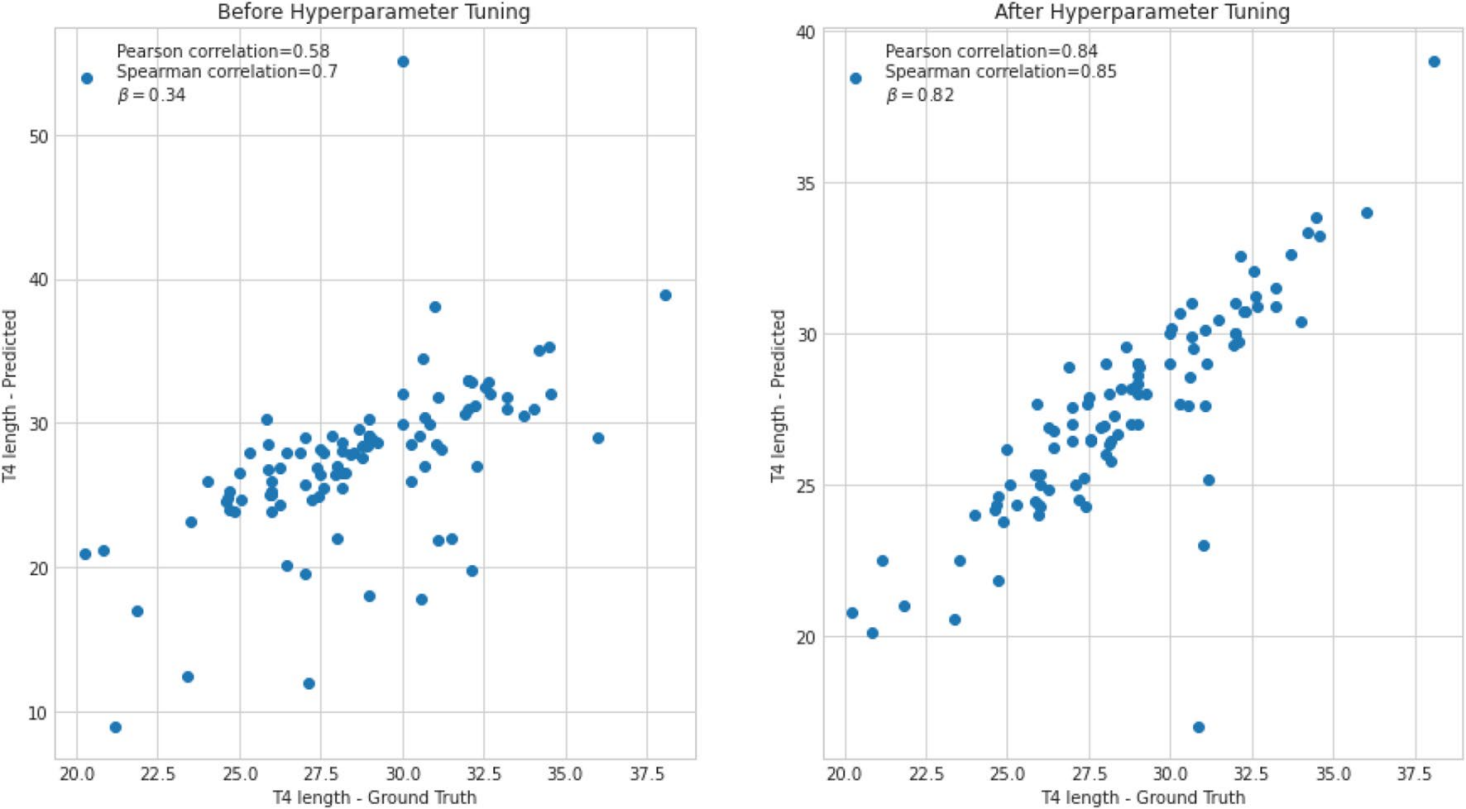
Scatter plots of ground truth T4 body length and predicted length by segmentation model. Pearson correlation coefficient, spearman correlation coefficient, and beta coefficient from linear regression for model with default hyperparameter setting (left) were 0.58, 0.7, 0.34, and 0.84, 0.85, 0.82 for model with tuned hyperparameters (right).

Dice score, precision, and recall values between the predicted and ground truth masks of 100 test images from each segmentation model are listed in Table 4. Predicted masks were converted to binary masks by applying a threshold of 0.5. We also investigated tuning the hyperparameters by comparing the segmentation performance between our new hyperparameter and default setting. The examples of segmentation outputs are listed in Figs. 4 and 5, and a comparison of accuracy in T4 body length measurement is shown in Fig. 6.

**Table 4:** Key performance indices of segmentation networks.

Type	Heart segmentation network		T4 body segmentation network	
Hyperparameter tuning	Before	After	Before	After
Hyperparameter α for Tversky Loss	0.5	0.7	0.5	0.2
Initial learning rate	0.01	0.001	0.01	0.001
Dice score coefficient	0.961	0.962	0.792	0.857
Recall	0.956	0.961	0.782	0.824
Precision	0.968	0.964	0.837	0.902

#### Performance of aHVI for detecting cardiomegaly

**Figure 7:**
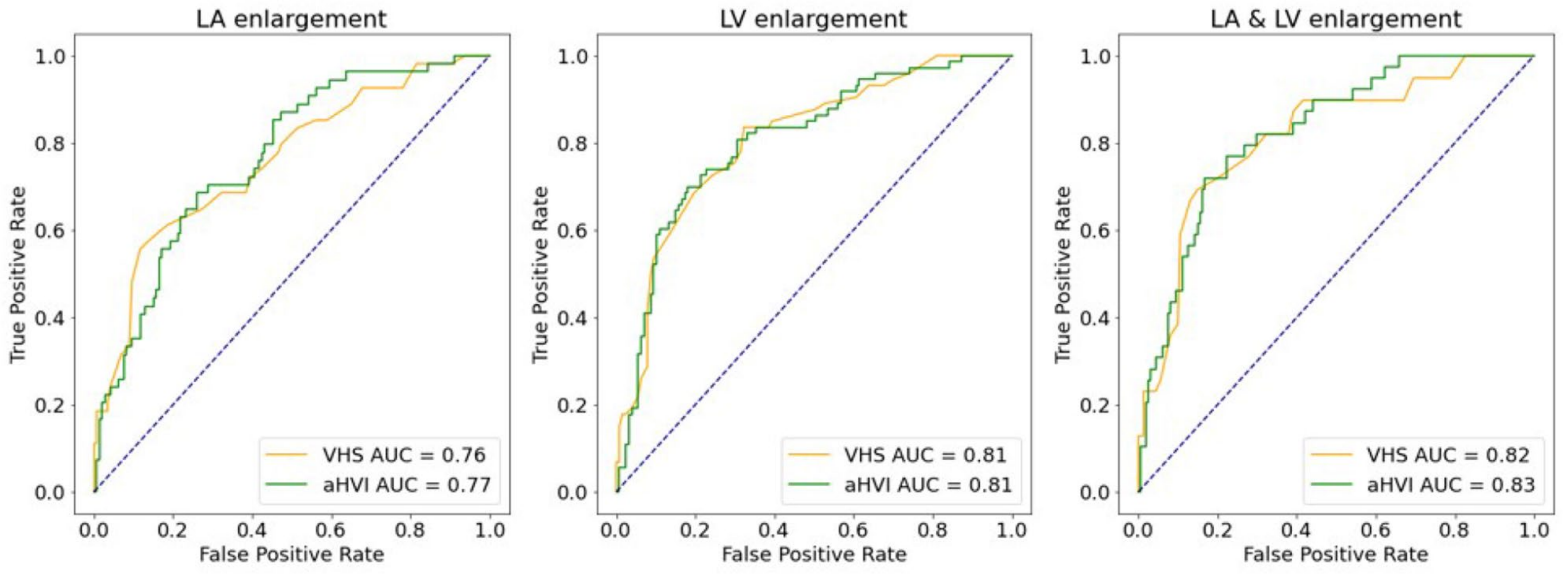
Area under the receiver operating characteristic (AUROC) curves of aHVI (green) and VHS (orange) in classifying left atrial enlargement (defined as LA/Ao < 1.6 and LA/Ao ≥ 1.6, left), left ventricular enlargement (defined as LVIDDN < 1.7 and LVIDDN ≥ 1.7, middle) and both left atrial and ventricular enlargement (defined as LA/Ao < 1.6 or LVIDDN < 1.7 and LA/Ao ≥ 1.6 and LVIDDN ≥ 1.7, right).

Calculated from the segmentation model, aHVI showed the mean value of 13.5 $\pm$ 4.7. The diagnostic performance of aHVI to detect left atrial enlargement, left ventricular enlargement, and combined left atrial/ventricular enlargement is shown in Fig. 7. The area under the curve of ROC (AUROC) for VHS and aHVI were respectively 0.76 (95% CI: 0.68, 0.84) and 0.77 (95% CI: 0.70, 0.84) in classifying left atrial enlargement, 0.81 (95% CI: 0.75, 0.87) and 0.81 (95% CI: 0.74, 0.89) in classifying left ventricular enlargement, 0.82 (95% CI: 0.74, 0.89) and 0.83 (95% CI: 0.76, 0.89) in classifying combined left atrial and ventricular enlargement. Sensitivity, specificity, F1 score values were calculated using an optimal cutoff value of VHS and aHVI. Cutoff value of VHS was 11.5, as guided^2^ (Table 5). We used Youden’s J statistic to calculate the cutoff value of aHVI, the cutoff value with the highest difference between true positive rate and false-positive rate. The cutoff was estimated from the left atrial enlargement classification task, with a value of 13.5.

**Table 5:** Prediction performance comparison between VHS and aHVI.

		Sensitivity	Specificity	F1 Score
Left Atrial Enlargement (LA/Ao ≥ 1.6)	VHS (cutoff = 11.5)	0.58	0.79	0.42
	aHVI (cutoff = 13.5)	0.67	0.74	0.56
Left Ventricular Enlargement (LVIDDN ≥ 1.7)	VHS (cutoff = 11.5)	0.68	0.69	0.40
	aHVI (cutoff = 13.5)	0.70	0.82	0.69
Left Atrial & Ventricular Enlargement (LA/Ao ≥ 1.6 & LVIDDN ≥ 1.7)	VHS (cutoff = 11.5)	0.48	0.86	0.43
	aHVI (cutoff = 13.5)	0.79	0.73	0.55

## Discussion

This study presents a fully automated deep learning algorithm to estimate a new cardiac index (aHVI) to predict canine cardiomegaly from a simple chest X-ray. In the veterinarian practice, morphological characteristics of the heart such as VLAS (Vertebral Left Atrial Score) and VHS are used to predict cardiomegaly and decide the needs for further echocardiographic examination^3,17^. However, the data for the predictive performance of these indices is limited^18,19^. Considering that simple radiographic diagnosis is the mainstay of screening measures for dogs, it conveys practical importance to developing radiography-based indices. Our findings suggest that VHS, the recommended heart index by ACVIM, does have acceptable performance in predicting cardiomegaly (AUROC 0.82). The aHVI showed very similar performance for each left atrial and ventricular indices and slightly outperformed the VHS for the gold-standard composite cardiomegaly index, LA:Ao/LVIDDN (AUROC 0.83).

We estimated that an aHVI = 13.5 has the best discriminative value as a single cut-off from the ROC curve. When we use the cut-off value of aHVI = 13.5, the aHVI showed generally increased sensitivity and F1 score at the cost of decreasing specificity. The increase in sensitivity of the aHVI compared to the VHS is noteworthy (0.48 for VHS versus 0.79 for aHVI), whereas the decrease in the specificity is relatively tiny (0.86 versus 0.73). Given the screening nature of the radiographic index, we believe indices with higher sensitivity are more important than those with higher specificity. The diagnosis of enlarged heart from simple radiography ensues further work-up and preventive medication that may help prevent fatal cardiomegaly and heart failure.

The VHS inevitably involves manual measures by trained personnel and is prone to measurement errors. For our data, the correlation coefficient of VHS was r = 0.902 when two veterinarian doctors independently measured 30 images. For the same data, we got the same result using our aHVI method, which makes the aHVI highly reproducible. The aHVI was developed using standard Python language-based algorithms, and it is available for developing standard application program interfaces (APIs), which will allow real-time diagnosis for canine cardiomegaly.

To our knowledge, this is the first study reporting a fully automated method of measuring a dog’s heart condition from simple radiography. Considering that the need for veterinarian health care is snowballing, our study will provide data and references for future related studies.

In this study, we attempted to develop a new volumetric heart index with allometric adjustment, and aHVI was first calculated by the product of area and length of heart, then divided by the length of the T4 vertebra. The current VHS is a one-dimensional measure, and the new aHVI is a proxy of volumetric measure. We think the measurement of the length of the heart may involve some uncertainties, whereas the measurement of the area is more reliable than the length.

For accurate segmentation, we employed a semantic segmentation model using a fully convolutional network to thoracic radiographs. This study's two central RoIs, e.g., heart and T4 body, are different in size and measurement unit. The strategy of tuning hyper-parameters in the semantic segmentation needs to be different accordingly. For example, the segmentation of the heart, with ovoid shapes and frequently blurred margins, showed good performance when the “α for Tversky Loss” was set higher, meaning more emphasis on sensitivity over specificity. On the other hand, for the segmentation of the T4 vertebral body with small and rectangular shapes, using lower α for Tversky Loss showed better performance, emphasizing specificity.

There were some limitations to our study. First, this study was based on 1,000 radiographic images to develop and validate the DL model. Moreorver, the threshold value of the aHVI is not validated for those breeds who has different thoracic conformation^20–22^. Although U-Net can reach excellent performance with less than 500 datasets^13^, a larger number of data may improve the predictive performance considering the diversity of dog breeds. Full list of breeds for entire dataset is described in Supplementary Table 1. When we examined the outliers in our DL model, we found that the image data lack any quality issues or particular clinical problems (Supplementary Figure S1, S2). We believe the errors stem from sub-optimal training that additional data may improve. Second, the echocardiogram measure of VHS, LA:Ao ratio, and LVIDDN values were reported by multiple radiologists, and we could not perform the test for reliability or validity of the gold-standard measures. Specialized veterinarian radiologists did all the reports, and we believe it is not likely that the non-differential interpersonal errors between the radiologists would have affected our findings. Third, the data (200 images) with both radiograph and echocardiogram tend to be derived from dogs with a higher risk of cardiomegaly. The possible selection of data toward more severe cases may have affected the predictive values, but it is not likely, that the comparisons between VHS and aHVI were affected differentially by this possible selection.

In conclusion, we developed a new method to diagnose cardiomegaly from a simple X-ray of dogs and demonstrated that the new method might outperform the current standard practice. Our method is also fully automated using a deep learning algorithm and less prone to human errors. With automated measures and higher sensitivity, the proposed method may contribute to diagnosing cardiomegaly for dogs at earlier stages.

## Supplementary Information

Supplementary Information.

## Data Availability

The data sets generated during and/or analyzed during the current study are not publicly available because they are property of the Seoul National University Veterinary Medicine Teaching Hospital but are available from the corresponding author on reasonable request.

## Abbreviations

VHS: Vertebra heart score
T4: 4Th thoracic vertebral body
MMVD: Myxomatous mitral valve disease
HE: Heart enlargement
LAE: Left atrial enlargement
LA/Ao: Left atrial: aorta ratio in the right-sided short axis view in early diastole
LVIDDN: Left ventricular internal diameter in diastole, normalized for body weight
DL: Deep learning
aHVI: Adjusted heart volume index
TL: Tversky loss

## Code availability

All source codes are available at https://github.com/JeongYeojin/aHVI.
