# Supplementary material for: An automated deep learning method and novel cardiac index to detect canine cardiomegaly from simple radiography
Source: Sci Rep. 2022 Aug 25;12:14494. doi: 10.1038/s41598-022-18822-4 (PMC9411130; doi:10.1038/s41598-022-18822-4)
Supplement: Supplementary file 1 — Supplementary Information. [file 41598_2022_18822_MOESM1_ESM.docx]

# **Supplementary Figures**


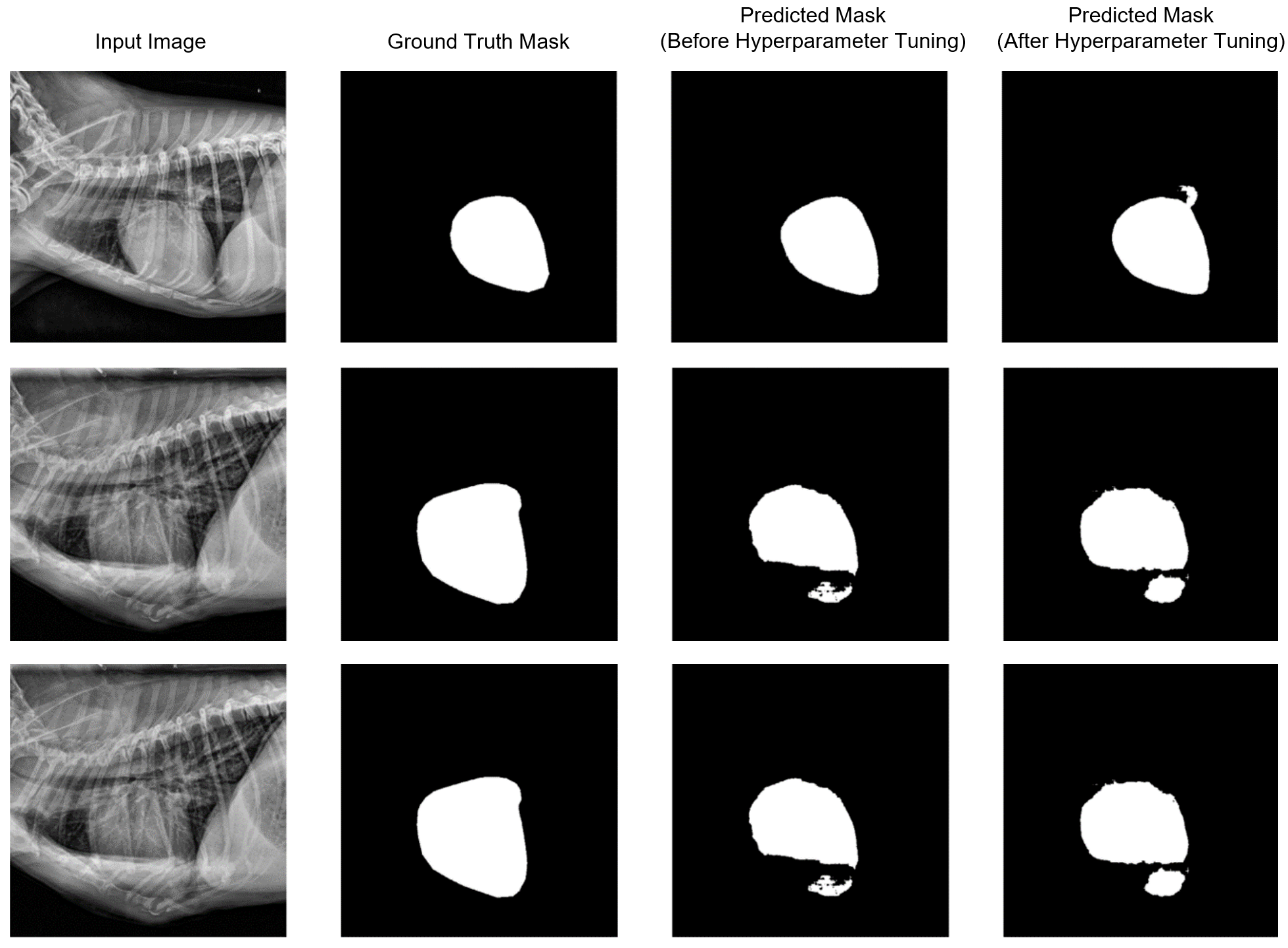


**Supplementary Figure S1.** Example of heart segmentation results with largest error

**
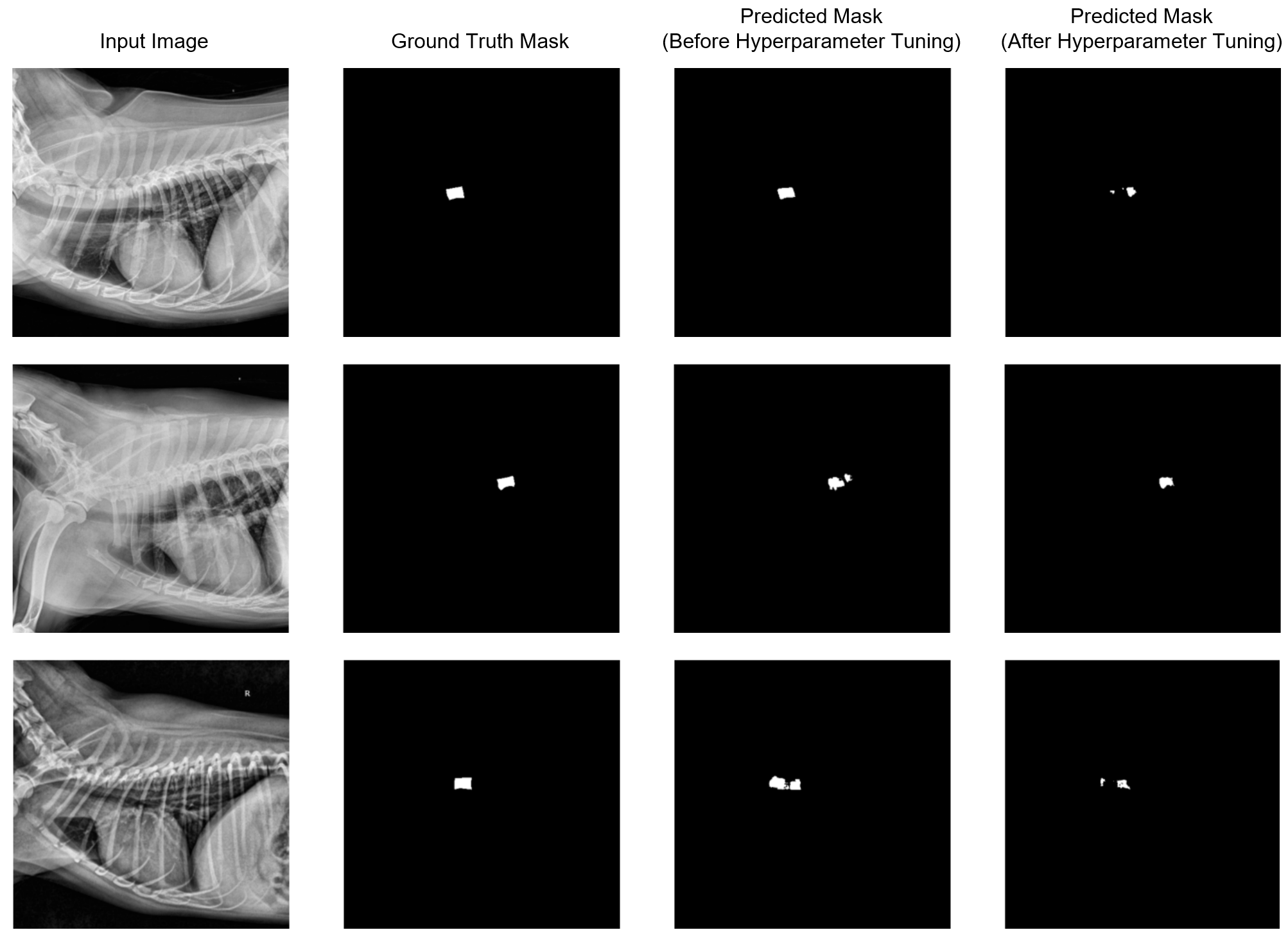
**

**Supplementary Figure S2.** Example of T4 segmentation results with largest error

# **Supplementary Tables**

Supplementary Table 1. List of breeds included in the study

| **Breed** | **DL Model Development Data (n=1000)** | **Echocardiography Validation Data (n=200)** |
| --- | --- | --- |
| Maltese | 192 | 59 |
| Shit Tzu | 125 | 35 |
| Poodle | 111 | 20 |
| Mix | 101 | 12 |
| Yorkshire Terrier | 57 | 11 |
| Cocker Spaniel | 58 | 5 |
| Pomeranian | 52 | 21 |
| Schnauzer | 29 | 1 |
| Dachshund | 25 | 1 |
| Chihuahua | 23 | 7 |
| Labrador Retriever | 23 | 4 |
| Golden Retriever | 19 | 1 |
| Bcihon Frice | 20 | 5 |
| Spitz | 17 | 7 |
| Pekinese | 14 | 3 |
| Jindo | 13 | 2 |
| Miniature Pinscher | 9 | 1 |
| Beagle | 7 | 1 |
| Shiba Inu | 6 | 0 |
| Welsh Corgi | 10 | 1 |
| Cavalier King Charles Spaniel | 5 | 0 |
| German Shepherd | 10 | 0 |
| Malinois | 4 | 0 |
| Shiverian Husky | 6 | 0 |
| Italian Greyhound | 6 | 0 |
| French Bulldog | 3 | 0 |
| White Terrier | 6 | 0 |
| Bedlington Terrier | 4 | 0 |
| Coton de tulear | 3 | 1 |
| Sapsaree | 3 | 0 |
| Sharpei | 4 | 0 |
| Scottish Terrier | 3 | 0 |
| Shetland Sheepdog | 3 | 1 |
| Pug | 2 | 0 |
| Afghan Hound | 2 | 0 |
| Pungsan Dog | 2 | 0 |
| Japanese Chin | 1 | 0 |
| Samoyed | 1 | 0 |
| Whippet | 1 | 0 |
| Malinois | 1 | 0 |
| Papilon | 1 | 0 |
| Chowchow | 2 | 0 |
| Jack Russell Terrier | 2 | 0 |
| Doberman Pinshcer | 1 | 0 |
| Boston Terrier | 1 | 0 |
| Alaskan Malamute | 1 | 0 |
| Standard Poodle | 1 | 0 |
| Rat Terrier | 1 | 0 |
| Old English Sheepdog | 1 | 0 |
| Weimaraner | 1 | 0 |
| Rottweirer | 1 | 0 |
| Fox Terrier | 1 | 0 |
| Italina Wirehaird Hound | 1 | 0 |
| Turkish Kangal | 1 | 0 |
| Border Collie | 1 | 0 |
| Old English Bulldog | 1 | 0 |
| Great Pyrenees | 1 | 1 |
| Total | 1000 | 200 |
